# NaCl Stress Stimulates Phenolics Biosynthesis and Antioxidant System Enhancement of Quinoa Germinated after Magnetic Field Pretreatment

**DOI:** 10.3390/foods13203278

**Published:** 2024-10-16

**Authors:** Shufang Wang, Xuejiao Zhang, Yiting Wang, Jirong Wu, Yin-Won Lee, Jianhong Xu, Runqiang Yang

**Affiliations:** 1 Jiangsu Key Laboratory for Food Quality and Safety/State Key Laboratory Cultivation Base, Ministry of Science and Technology/Key Laboratory for Control Technology and Standard for Agro-Product Safety and Quality, Ministry of Agriculture and Rural Affairs/Key Laboratory for Agro-Product Safety Risk Evaluation (Nanjing), Ministry of Agriculture and Rural Affairs/Collaborative Innovation Center for Modern Grain Circulation and Safety/Institute of Food Safety and Nutrition, Jiangsu Academy of Agricultural Sciences, Nanjing 210014, China; wangshufang202301@163.com (S.W.); yangzhouwjr@126.com (J.W.); lee2443@snu.ac.kr (Y.-W.L.); 2 College of Food Science and Technology, Whole Grain Food Engineering Research Center, Nanjing Agricultural University, Nanjing 210095, China; zhangxuejiao8@126.com (X.Z.); 9201810401@stu.njau.edu.cn (Y.W.); 3 Department of Agricultural Biotechnology, Seoul National University, Seoul 08826, Republic of Korea

**Keywords:** *Chenopodium quinoa*, NaCl stress, germinated quinoa growth, magnetic field pretreatment, phenolic compounds, flavonoids, antioxidant activity

## Abstract

Our previous study showed that magnetic field pretreatment promoted germination and phenolic enrichment in quinoa. In this study, we further investigated the effects of NaCl stress on the growth and phenolic synthesis of germinated quinoa after magnetic field pretreatment (MGQ). The results showed that NaCl stress inhibited the growth of MGQ, reduced the moisture content and weight of a single plant, but increased the fresh/dry weight. The higher the NaCl concentration, the more obvious the inhibition effect. In addition, NaCl stress inhibited the hydrolysis of MGQ starch, protein, and fat but increased the ash content. Moreover, lower concentrations (50 and 100 mM) of NaCl stress increased the content of MGQ flavonoids and other phenolic compounds. This was due to the fact that NaCl stress further increased the enzyme activities of PAL, C4H, 4CL, CHS, CHI, and CHR and up-regulated the gene expression of the above enzymes. NaCl stress at 50 and 100 mM increased the DPPH and ABTS scavenging capacity of MGQ and increased the activities of antioxidant enzymes, including SOD, POD, CAT, APX, and GSH-Px, further enhancing the antioxidant system. Furthermore, principal component analysis showed that NaCl stress at 100 mM had the greatest combined effect on MGQ. Taken together, NaCl stress inhibited the growth of MGQ, but appropriate concentrations of NaCl stress, especially 100 mM, helped to further increase the phenolic content of MGQ and enhance its antioxidant system.

## 1. Introduction

Quinoa (*Chenopodium quinoa* Willd.) is a pseudo-cereal crop rich in nutrients and known for its high-quality protein and essential amino acid content. As a result, the Food and Agriculture Organization (FAO) has classified it as one of the top ten most healthful and nourishing foods for global human consumption [1]. Quinoa also contains numerous functional components, including polyphenols and flavonoids. These substances have many potential health benefits, and their sustained intake can aid in the prevention and treatment of various diseases, such as inflammation, obesity, diabetes, and cancer [2]. Moreover, quinoa is particularly resistant to extreme environmental conditions, including drought, salt, and frost [3]. Consequently, quinoa has increasingly become a keen subject of research in recent years.

Germination is a plant’s most active growth stage in which macromolecules such as starch, proteins, and lipids in edible seeds are converted into smaller molecules such as amino acids and reducing sugars that the body can absorb more easily [4]. Germination not only improves the nutritional value and functional activity of quinoa, but it also provides the advantages of simplicity, safety, and environmental friendliness [5]. Nevertheless, germination is accompanied by the drawbacks of a lengthy duration and lack of uniformity. Hence, the methods that facilitate quinoa germination are highly intriguing. In our earlier study, we found that applying a magnetic field of 10 mT for 10 min as an external physical stimulus promoted the germination of quinoa [6]. This stimulus facilitated the conversion of macromolecules into smaller molecules and accelerated the provision of energy. Furthermore, the magnetic field increased the concentration of quinoa’s phenolic compounds and enhanced the antioxidant system by activating phenolic synthesis.

Salinity is a common abiotic stress in plant growth due to natural circumstances. Quinoa is regarded as one of the most salt-tolerant crops, having a tolerance range of 75 to 150 mM [7]. Although quinoa is able to adapt to salt environments, it is still sensitive to salt stress during the germination and sprout stages [8]. Excessive salt affects cellular activity, accumulates reactive oxygen species (ROS), and reduces water potential, resulting in osmotic stress that ultimately prevents normal plant growth [9]. Two quinoa cultivars, Hualhuas and Real, exhibited growth inhibition in response to high salinity treatment [10]. Salt stress inhibited the growth of quinoa sprouts during germination [11]. Salt stress also impeded quinoa germination by inhibiting starch hydrolysis to reducing sugars [12]. However, salt stress at appropriate concentrations increases phenolic compounds and the antioxidant activity of plants. For example, salt stress increased the phenolic content of quinoas as well as the scavenging capacity of DPPH and ABTS [13]. The antioxidant activity and phenolic and flavonoid content of buckwheat were enhanced when subjected to 100 mM NaCl stress [14]. Salt stress activated phenylpropane metabolism in strawberries by up-regulating gene expression of *FaPAL*, *FaC4H*, and *Fa4CL*, which promoted the synthesis of phenolic compounds [15].

Our initial research has demonstrated that a 10 mT magnetic field exposure for 10 min significantly promotes the germination, growth, and phenolic compound synthesis and strengthens the antioxidant system in quinoa [6]. The impact of subsequent treatments with varying concentrations of NaCl on these quinoa parameters has not been elucidated. It is hypothesized that the concurrent application of magnetic fields and NaCl may elicit synergistic effects, further augmenting phenolic biosynthesis and the antioxidant system in plants. However, there is a paucity of research on the combined effects of magnetic fields and NaCl on seed physiology, and the intricate mechanisms underlying these interactions remain largely unexplored.

This study aimed to (1) investigate the effects of NaCl stress at different concentrations on the growth characteristics and the degree of hydrolysis of macromolecules in MGQ; (2) investigate the effect and mechanism of NaCl stress on phenolic synthesis; and (3) examine the effect of NaCl stress on the antioxidant system. This work provides guidance for further developing and utilizing quinoa resources in the food industry by combining magnetic fields with NaCl stress.

## 2. Materials and Methods

### 2.1. Raw Materials and Reagents

Quinoa Sanjiang-1 (*Chenopodium quinoa*; SJ-1) seeds were provided by Dr. Xiaofeng Sun from Qinghai Academy of Agriculture and Forestry Sciences, Xining, Qinghai, China in 2022. Dried seeds were stored at −20 °C before use. All chemicals and reagents used were of analytical grade. The chemicals 1,1-diphenyl-2-trinitrophenylhydrazine (DPPH), 2,2-diazo-bis (3-ethyl-benzothiazole-6-sulfonic acid) diammonium salt (ABTS), and Trolox were purchased from Sigma-Aldrich Chemical Company (St. Louis, MO, USA). Mercaptoethanol, acetonitrile, and methanol (HPLC grade) were purchased from Maclean Biotechnology Co., Ltd. (Shanghai, China). Other chemicals and reagents used were of analytical grade.

### 2.2. Materials’ Treatment Methods

Impurities were removed from plump and undamaged SJ-1 quinoa seeds by sieving. The seeds were immersed in 1% sodium hypochlorite solution for 10 min before being rinsed with deionized water to remove any remaining sodium hypochlorite from the surface of the quinoa. After soaking in deionized water at 30 °C for 3 h, quinoa seeds were treated with a magnetic field strength of 10 mT for 10 min [6]. The corresponding weights of NaCl were weighed separately and configured into 0, 50, 100, 200, and 300 mM NaCl solutions using distilled water. Then, the magnetic field-treated quinoa seeds were uniformly sown in plastic trays containing different concentrations of the NaCl culture solution. Additionally, the NaCl solution was sprayed every hour for 2 min each time, and the NaCl culture solution was changed once a day. The quinoa seeds were then germinated under different NaCl stresses (0, 50, 100, 200, and 300 mM) for 24 h (30 °C, RH 80%) and recorded as M+N0, M+N50, M+N100, M+N200, and M+N300, respectively. The schematic diagram depicting the device used for quinoa treatment and germination is illustrated in Fig. S1. Control seeds were subjected to germination under the same conditions after magnetic field pretreatment but without NaCl treatment. The germinated quinoa obtained was freeze dried and stored for further analysis.

### 2.3. Determination of Sprout Length, Germination Percentage, Single Plant Weight, Moisture Content, and Fresh/Dry Weight

The sprout length was determined using vernier calipers. The germination percentage was determined by randomly selecting 30 seeds from each treatment group. The single plant weight was determined by randomly selecting 30 sprouted quinoas from each treatment group. The fresh weight (FW) was calculated by weighing 100 sprouted quinoa seeds chosen at random from each treatment group. The weight of these sprouted quinoas after oven drying was then calculated as the dry weight (DW). The fresh/dry weight was calculated by dividing the fresh weight by the dry weight. The moisture content (%) was estimated as (FW-DW)/FW × 100 [16].

### 2.4. Determination of Starch, Reducing Sugar, Soluble Protein, Free Amino Acid, Crude Fat, and Ash Content

The starch content was measured using the iodine colorimetric method [17]. First, 0.1 g of the sample was washed with 10 mL of 80% ethanol to remove alcohol-soluble impurities. The centrifuged residue was then rinsed with 10 mL of distilled water to eliminate water-soluble impurities, centrifuged again, and suspended in 10 mL of an 80% Ca(NO_3_)_2_ solution. The supernatant was then collected after 10 min in a hot water bath. Finally, 0.1 mL of the supernatant was reacted with I_2_, and the absorbance was measured at 620 nm.

The reducing sugar content was determined using the dinitrosalicylic acid (DNS) method [18]. A mixture of 0.3 g of sample and 5 mL of distilled water was centrifuged at 10,000× *g* for 10 min after being heated at 50 ℃ for 20 min. The residue was rinsed with 2 mL of distilled water and centrifuged. The supernatant was volume-filled to 10 mL with distilled water. Then, 2 mL of the supernatant was mixed with 1.5 mL of the DNS solution and reacted in boiling water for 5 min, then cooled. The absorbance was recorded on an HP 33120A UV–vis spectrophotometer (Feile Instrument Co., Ltd., Shanghai, China) at 540 nm. A standard curve was obtained using glucose as the standard.

The soluble proteins were determined using the Bradford method [19]. One gram of sample was accurately weighed and dissolved in 20 mL of ultrapure water, sonicated, and centrifuged (10,000× *g*, 10 min). The supernatant was collected and diluted to 50 mL with water for measurement. The Coomassie Brilliant Blue G250 solution was made by mixing 50 mL of ethanol (90%) and 50 mL of phosphoric acid (85%) and diluting to 1000 mL with ultrapure water. The solution to be tested (0.5 mL), ultrapure water (1.5 mL), and Coomassie Brilliant Blue G250 (5 mL) were mixed in a water bath and heated at 30 °C for 10 min. The absorbance value was measured at 595 nm and estimated by constructing a standard curve with bovine serum protein.

The free amino acid content was measured with an amino acid analyzer. First, 0.03 g of sample was mixed with 6 M HCL in a closed container and filled with nitrogen for acid hydrolysis at 110 °C for 24 h. The mixture was chilled, filtered, and volume-fixed with ultrapure water to 50 mL. After drying with nitrogen, 2 mL of the material was dissolved in 0.02 M HCl and centrifuged at 3000× *g* for 15 min. The supernatant was examined using a syringe through a 0.22 μm microporous filter membrane [20].

The crude fat and ash contents were determined using AOAC standard methods 960.39 and 923.03, respectively [21].

### 2.5. Determination of Phenolic and Flavonoid Content

Both free and bound phenolic compounds were extracted using the methods described by Ti et al. [22] and Sun et al. [23]. A 0.5 g sample was homogenized by adding 5 mL of pre-cooled acetone solution on ice, and the supernatant was centrifuged at 4 °C and 10,000× *g* for 10 min. The collected supernatant was concentrated under vacuum at 45 °C before being reconstituted with distilled water to 10 mL for measurement. After free phenol extraction, the residue was hydrolyzed with 40 mL of 2 M NaOH under nitrogen gas at room temperature for 1 h. The residue was then neutralized with concentrated hydrochloric acid to pH 2 before being extracted with ethyl acetate five times. The mixture was then concentrated and dried under a vacuum at 45 °C before being redissolved in 10 mL of distilled water for measurement.

According to the method of Kim et al. [24], 0.3 mL of a 5% sodium nitrite solution was mixed with 1 mL of the free sample solution and 4 mL of the bound sample solution for 6 min. Afterwards, 0.3 mL of a 10% aluminum nitrate solution was added, and the mixture was mixed again for 6 min. Next, 4 mL of a 4% sodium hydroxide solution was added and mixed. The final volume was fixed to 10 mL with 80% ethanol, and the content of phenolics and flavonoids was determined by measuring the absorbance values at 760 nm and 510 nm, respectively, after standing for 15 min. The free and bound phenolic contents were calculated using the gallic acid standard curve, and the free and bound flavonoid contents were calculated using rutin as the standard.

### 2.6. Determination of Key Enzyme Activity in the Phenolic Synthesis Pathway

The activity of phenylalanine ammonia-lyase (PAL) was assessed using the method described by Assis et al. [25]. The cinnamate 4-hydroxylase (C4H) activity assay was performed using the method of Liu et al. [26] with slight modifications. A precisely weighed 0.5 g sample was mixed with a phosphate buffer (5 mL, pH 7.6, 100 mM), and then placed on ice for homogenization. Afterwards, it was subjected to centrifugation at 4 °C and 10,000× *g* for 10 min. Subsequently, 0.1 mL of the supernatant was taken and reacted with trans-cinnamic acid (50 mM), nicotinamide adenine dinucleotide phosphate (2 mL, 0.5 M), and a phosphate buffer (3 mL, 100 mM) at 30 °C for 60 min, and then the absorbance was measured at 340 nm. The 4-coumarate: coenzyme A ligase (4CL) activity was determined using the method described by Ma et al. [27]. The activities of chalcone synthase (CHS), chalcone reductase (CHR), and chalcone isomerase (CHI) were determined with an ELISA kit purchased from Nanjing Jiancheng Bioengineering Research Institute.

### 2.7. Determination of Key Enzyme Gene Expression in Phenolic Synthesis Pathway

Gene expression was measured using a fluorescent quantitative PCR instrument (QuantStudioR5, Fisher Scientific, Shanghai, China) and SYBR Premix Ex TaqTM (Takara, Beijing, China; catalog No. RR420A). The primers were designed using Primer 5.0, and relative gene expression was determined with the 2^−ΔΔCt^ method. The primer sequences are shown in Table 1.

### 2.8. Determination of Antioxidation Capacity

The DPPH and ABTS scavenging capacities were determined by using the kits from Suzhou Keming Biotechnology Co. The samples were precisely weighed at 0.1 g and added to 1 mL of extraction solution. The mixture was homogenized on ice and then centrifuged at 4 °C and 10,000× *g* for 10 min to collect the supernatant for measurement. The absorbance values at wavelengths of 515 nm and 734 nm were measured following addition of the reagents according to the instructions.

### 2.9. Determination of Antioxidative Enzyme Activity

Peroxidase (POD) activity was determined according to the method of Jin et al. [28]. The activities of catalase (CAT), superoxide dismutase (SOD), ascorbate peroxidase (APX), and glutathione-peroxidase (GSH-Px) were determined using kits from the Nanjing Jiancheng Bioengineering Institute.

### 2.10. Statistical Analysis

All assays were performed with three biological replicates. Data processing and statistical analyses were conducted using GraphPad Prism 7. The data are presented as mean ± standard deviation (±SD). One-way analysis of variance (ANOVA) was used to assess differences between treatments. Different lowercase letters represent statistically significant differences between treatment groups (*p* < 0.05). Principal component analysis was performed in SPSS 26.0 using dimensionality reduction factors.

## 3. Results

### 3.1. Effect of NaCl Stress on the Morphology of Quinoa Sprouts after Magnetic Field Pretreatment

**Figure 1:**
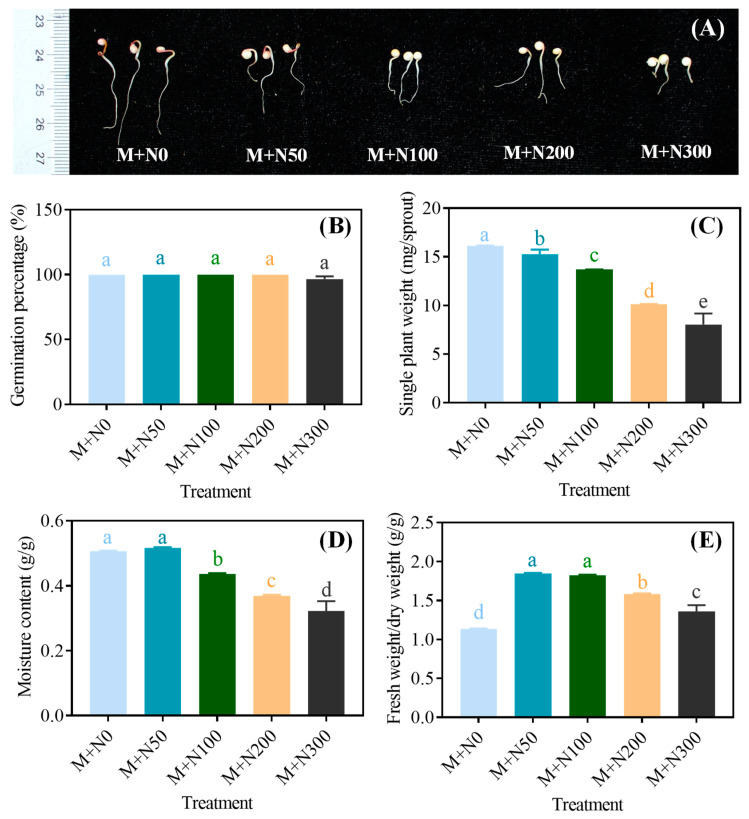
Effects of NaCl stress on the sprout length (**A**), germination percentage (**B**), single plant weight (**C**), moisture content (**D**), and fresh weight/dry weight (**E**) of germinated quinoa after magnetic field pretreatment. Values are expressed as mean ± SD. Lowercase letters represent significant differences among treatments (*p* < 0.05).

The growth of MGQ was inhibited by NaCl stress, with a greater severity of NaCl stress resulting in a more significant inhibition (Figure 1A). NaCl stress had no significant effect on germination percentage of MGQ; only the M+N300 group exhibited a slightly reduced germination percentage compared to the other groups (Figure 1B). All NaCl stressors reduced the single plant weight of MGQ. The M+N50 and M+N300 groups had lower single plant weights than the M+N0 group by 5.5% and 50.3%, respectively (Figure 1C). NaCl stress reduced MGQ moisture content, with the exception of the M+N50 group. The M+N100, M+N200, and M+N300 groups had lower moisture content than that of the M+N0 group by 13.9%, 27.3%, and 36.3%, respectively (Figure 1D). NaCl stress increased the fresh/dry weight of MGQ, which tended to increase and then decrease as NaCl concentration increased. The M+N50 and M+N100 groups exhibited respective increases of 62.9% and 61.0% in comparison to the M+N0 group (Figure 1E).

### 3.2. Effect of NaCl Stress on the Basic Nutrient Content of Quinoa Sprouts after Magnetic Field Pretreatment

**Figure 2:**
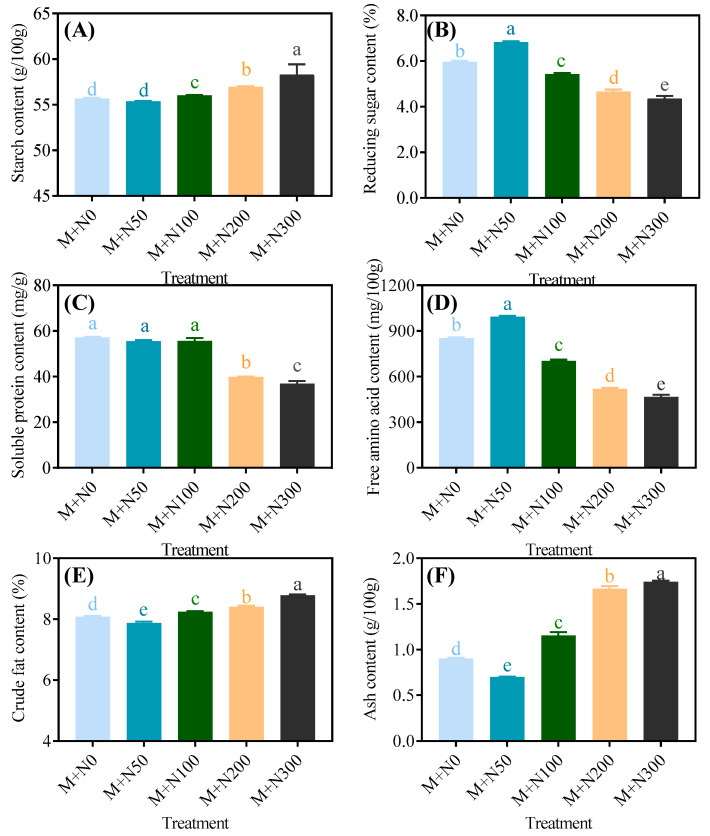
Effects of NaCl stress on the starch (**A**), reducing sugar (**B**), soluble protein (**C**), free amino acid (**D**), crude fat (**E**), and ash (**F**) content of germinated quinoa after magnetic field pretreatment. Values are expressed as mean ± SD. Lowercase letters represent significant differences among treatments (*p* < 0.05).

With the exception of the M+N50 group, NaCl stress increased the starch content of MGQ. As the concentration of NaCl increased, the starch content also increased. The M+N300 group had a starch content that was 4.7% higher than the M+N0 group (Figure 2A). The M+N50 group had a 14.5% higher reducing sugar content than the M+N0 group, while all of the other groups had a lower reducing sugar content (Figure 2B). The soluble protein content of the M+N50 and M+N100 groups was slightly lower than that of the M+N0 group, whereas the M+N200 and M+N300 groups were less than 30.3% and 35.55%, respectively (Figure 2C). NaCl stress reduced the free amino acid content of MGQ, with the exception of the M+N50 group. The M+N100, M+N200, and M+N300 groups had lower free amino acid content than the M+N0 group by 17.59%, 39.0%, and 45.6%, respectively (Figure 2D). NaCl stress increased the fat content of MGQ, with the exception of the M+N50 group. The greater the NaCl stress, the higher the fat content (Figure 2E). The ash content of the M+N50 group was lower than that of the M+N0 group by 22.4%, whereas all other groups were higher than that of the M+N0 group, and the higher the NaCl concentration, the greater the ash content (Figure 2F).

### 3.3. Effect of NaCl Stress on the Phenolic Content of Quinoa Sprouts after Magnetic Field Pretreatment

**Figure 3:**
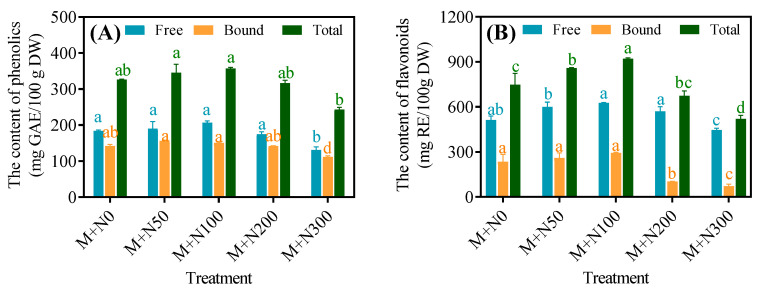
Effects of NaCl stress on the phenolics (**A**) and flavonoids (**B**) content of germinated quinoa after magnetic field pretreatment. Values are expressed as mean ± SD. Lowercase letters represent significant differences among treatments (*p* < 0.05).

The phenolic content of MGQ increased and then decreased as the NaCl concentration increased, with the M+N100 group having the highest free and total phenol levels, which were 12.2% and 9.6% greater, respectively, than the M+N0 group (Figure 3A). Similarly, the flavonoid content increased and then decreased as the NaCl concentration increased. The levels of free flavonoids, bound flavonoids, and total flavonoids reached their maximum in the M+N100 group, surpassing those of the M+N0 group by 22.2%, 24.9%, and 23.0%, respectively (Figure 3B).

### 3.4. Effect of NaCl Stress on the Activity and the Gene Expression of Phenolic Synthase in Quinoa Sprouts after Magnetic Field Pretreatment

#### 3.4.1. Enzyme Activity

**Figure 4:**
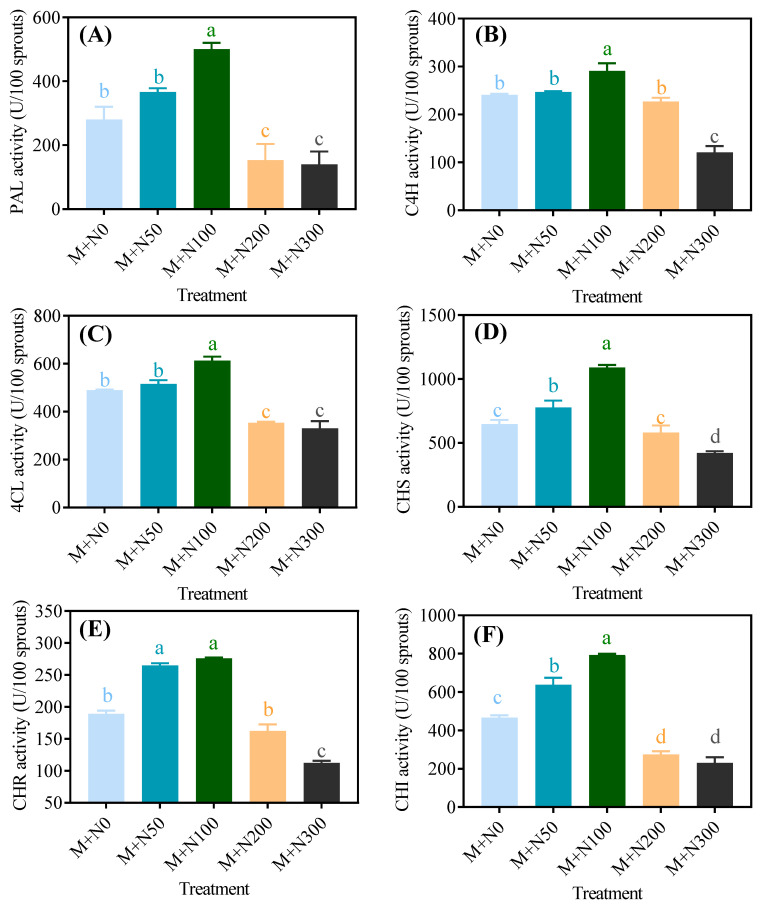
Effects of NaCl stress on the activities of PAL (**A**), C4H (**B**), 4CL (**C**), CHS (**D**), CHR (**E**), and CHI (**F**) of germinated quinoa after magnetic field pretreatment. Values are expressed as mean ± SD. Lowercase letters represent significant differences among treatments (*p* < 0.05).

As shown in Figure 4, the enzyme activities involved in phenolic production in MGQ exhibited a single-peak pattern, with all of them reaching their highest value at a NaCl concentration of 100 mM. The enzyme activities decreased as the NaCl concentration continued to increase. Moreover, increased NaCl concentrations resulted in reduced enzyme activity. The activities of PAL, C4H, 4CL, CHS, CHR, and CHI in the M+N100 group exceeded those in the M+N0 group by 78.6%, 20.9%, 25.1%, 68.31%, 45.8%, and 70.0%, respectively. The activities of PAL, C4H, 4CL, CHS, CHR, and CHI in the M+N300 group increased by 50.0%, 49.7%, 32.6%, 34.7%, 40.7%, and 50.7% compared to the M+N0 group, respectively.

#### 3.4.2. Gene Expression

**Figure 5:**
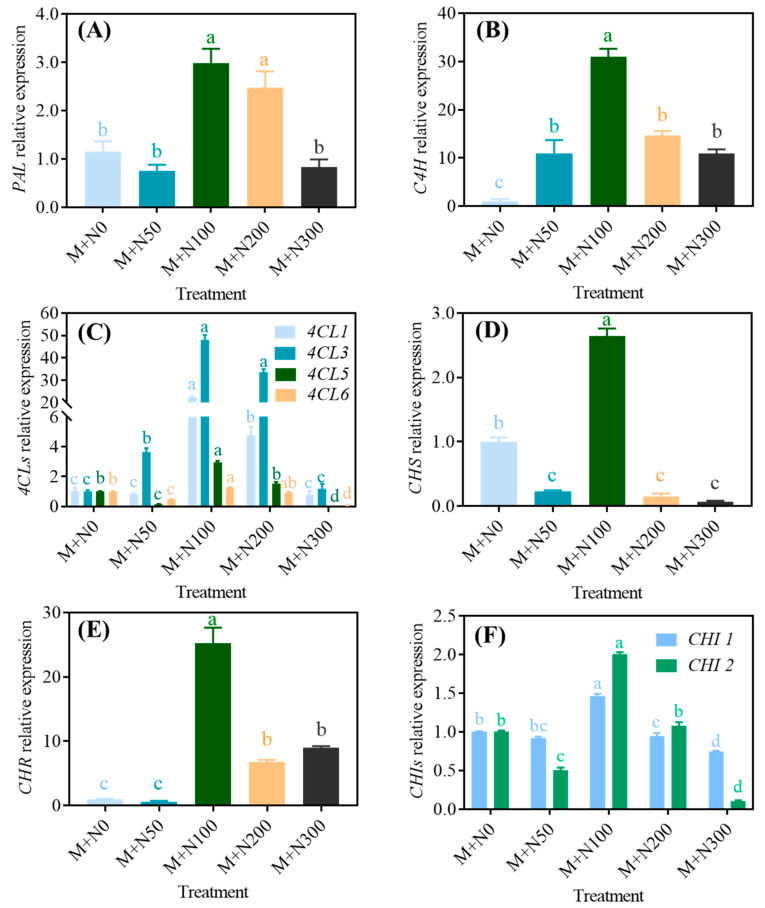
Effects of NaCl stress on the gene expression of *PAL* (**A**), *C4H* (**B**), *4CLs* (**C**), *CHS* (**D**), *CHR* (**E**), and *CHIs* (**F**) of germinated quinoa after magnetic field pretreatment. Values are expressed as mean ± SD. Lowercase letters represent significant differences among treatments (*p* < 0.05).

As shown in Figure 5, the expressions of phenolic-synthesis-related genes exhibited their highest levels in the group treated with 100 mM NaCl. These levels were significantly greater than those observed in the M+N0 group. With the exception of *C4H* and *4CL2*, the expression of all other genes in the M+N50 group was comparatively lower than that in the M+N0 group. *C4H* and *4CL2* gene expression in the M+N50 group was 9.42-fold and 2.62-fold greater than compared to the M+N0 group. Except for *4CL4*, *CHS*, and *CHI1*, all other genes in the M+N200 group showed higher levels than those in the M+N0 group. Except for *CHR*, the M+N300 group was lower than the M+N200 group.

### 3.5. Effect of NaCl Stress on the Antioxidative System of Quinoa Sprouts after Magnetic Field Pretreatment

#### 3.5.1. Antioxidative Capacity

**Figure 6:**
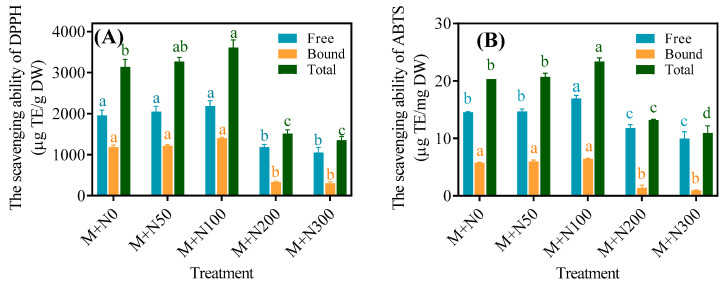
Effects of NaCl stress on the scavenging capacity of DPPH (**A**) and ABTS (**B**) of germinated quinoa after magnetic field pretreatment. Values are expressed as mean ± SD. Lowercase letters represent significant differences among treatments (*p* < 0.05).

The DPPH scavenging capability of free phenols, bound phenols, and total phenols exhibited a pattern of initially increasing and then decreasing with the increase in NaCl concentration. The M+N50 and M+N100 groups exhibited a greater DPPH scavenging capability compared to the M+N0 group, while the M+N200 and M+N300 groups had a lower scavenging capability than the M+N0 group. The DPPH scavenging capability of free phenols, bound phenols, and total phenols in the M+N100 group was higher than that in the M+N0 group by 11.6%, 18.4%, and 14.9%, respectively (Figure 6A). The ABTS scavenging capability of free phenols, bound phenols, and total phenols exhibited comparable trends to those for DPPH. The ABTS scavenging capability of free phenols, bound phenols, and total phenols in the M+N100 group was 16.6%, 11.1%, and 15.0% greater, respectively, than that in the M+N0 group (Figure 6B).

#### 3.5.2. Antioxidative Enzyme Activity

**Figure 7:**
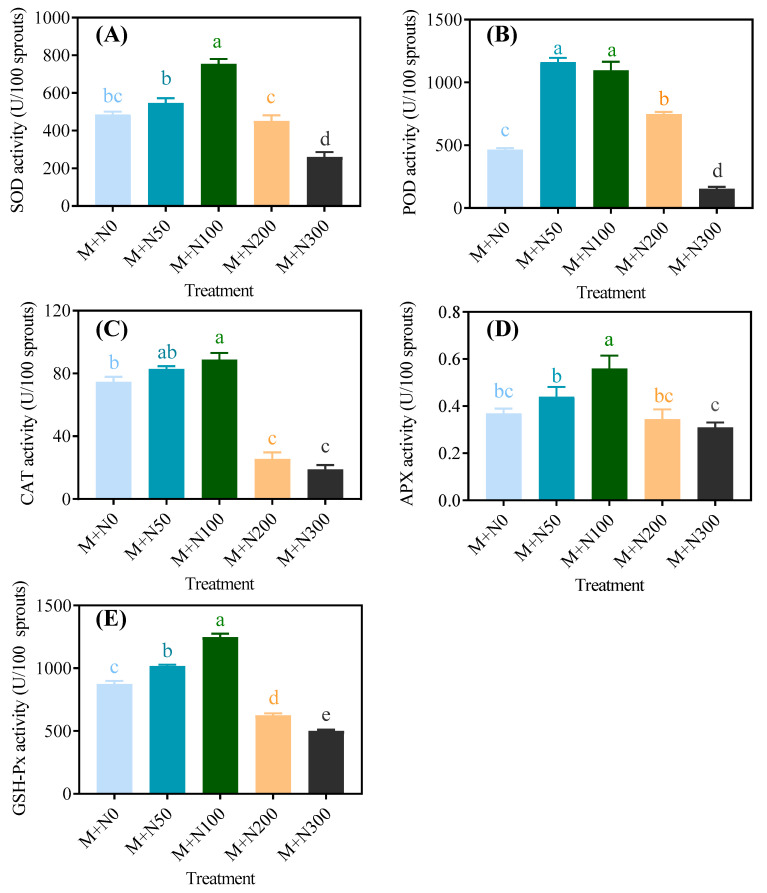
Effects of NaCl stress on the antioxidative enzyme activity of SOD (**A**), POD (**B**), CAT (**C**), APX (**D**), and GSH-Px (**E**) of germinated quinoa after magnetic field pretreatment. Values are expressed as mean ± SD. Lowercase letters represent significant differences among treatments (*p* < 0.05).

As shown in Figure 7, the enzyme activities of SOD, POD, CAT, APX, and GSH-Px in MGQ exhibited a consistent pattern of change. They all initially increased and then dropped as the NaCl concentration increased. The enzyme activity of POD reached its peak in the M+N50 group, while the activities of other enzymes reached their peak in the M+N100 group. The enzyme activities of SOD, CAT, APX, and GSH-Px were 55.4%, 19.0%, 51.6%, and 42.7% greater, respectively, in the M+N100 group compared to the M+N0 group. In addition, with the exception of POD, the other antioxidant enzyme activities of germinated quinoa were inhibited when exposed to NaCl concentrations exceeding 100 mM.

### 3.6. Principal Component Analysis (PCA)

**Figure 8:**
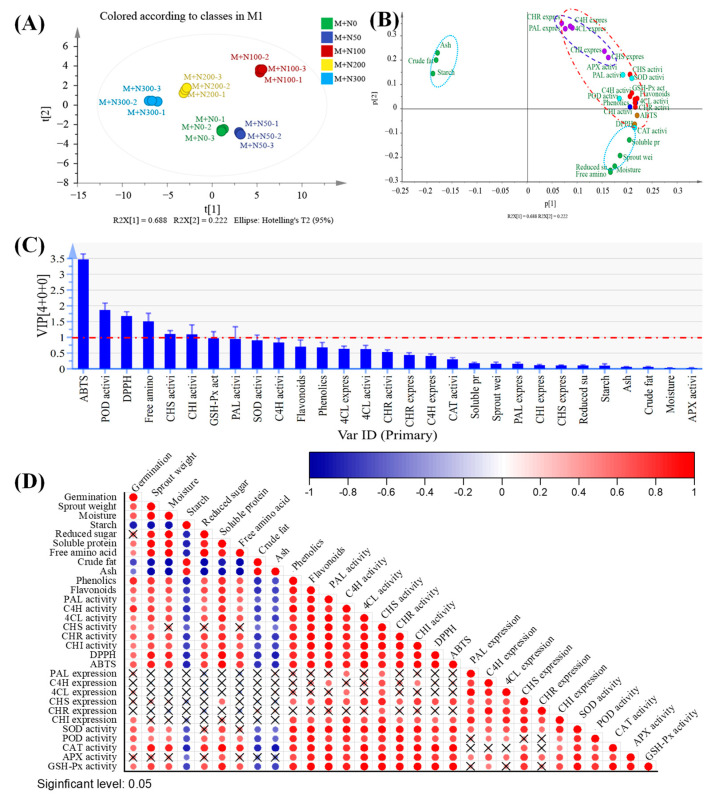
A comprehensive evaluation of the effects of various NaCl concentrations on germinated quinoa following pretreatment with a magnetic field (**A**). Rotated component matrix of principal component analysis (**B**). Indicators of significant differences under NaCl stress (**C**). Correlation analysis of indices of germinated quinoa after magnetic field pretreatment under different NaCl concentrations (**D**).

PCA was employed to comprehensively evaluate the indicators of MGQ under NaCl stress. The total scores for each NaCl stress treatment are displayed in Figure 8A. The results demonstrate that 100 mM NaCl had a significant effect on MGQ. Three principal components with eigenvalues greater than 1 were selected, and the loading matrices of the first three principal components are shown in Figure 8B. The ABTS and DPPH scavenging capabilities and POD enzyme activity were the most significant indicators of variations in the MGQ under NaCl stress (Figure 8C). The correlation analysis revealed a positive relationship between germination percentage and single plant weight with moisture, reducing sugar, soluble protein, and free amino acid content. Conversely, there was a negative correlation with starch, total fat, and ash content. The levels of phenolic and flavonoid contents were positively correlated with the enzyme activities of PAL, C4H, 4CL, CHS, CHI, and CHR. Furthermore, there was a positive correlation between the phenolic content and the antioxidant capacity as well as the activities of antioxidant enzymes such as SOD, POD, CAT, APX, and GSH-Px (Figure 8D).

## 4. Discussion

Salt stress has become one of the major abiotic stresses limiting plant growth. Salt stress reduces seed swelling, thus inhibiting seed germination [29]. Furthermore, high salinity can disrupt the dynamic balance of water potential and ion distribution in plant cells, inhibiting plant growth [30]. In this study, we found that NaCl stress did not affect the germination rate of quinoa. This could be attributed to the pretreatment of quinoa with a magnetic field, which stimulated germination prior to application of NaCl stress and thereby alleviated a portion of NaCl stress. However, NaCl stress reduced the moisture content of MGQ, resulting in a decrease in both the single plant weight and the length of the sprouts. The inhibitory impact became more pronounced as the concentration of NaCl increased (Figure 1). This is comparable in part to the finding of [31], which reported that NaCl stress decreased water content and single plant height in Leaf Beet. Salt stress also caused a remarkable accumulation of reactive oxygen species (ROS) in the plant cells, resulting in the disruption of the integrity of the cell membrane [32]. As a result, NaCl stress may cause oxidative damage by producing excessive ROS and inhibiting the sprout growth of MGQ. During the seed germination stage, starch, proteins, and lipids are hydrolyzed into small molecules such as reducing sugars and amino acids, which provide energy for plant growth and development [33]. In this study, high levels of NaCl stress inhibited the hydrolysis of starch to reducing sugars, protein to amino acids, and lipids to fatty acids in MGQ (Figure 2). This result is partially similar to that of salt stress inhibiting the hydrolysis of quinoa starch to soluble sugars [12]. Nevertheless, NaCl stress at 50 mM stimulated the hydrolysis of starch, protein, and lipid. We hypothesize that this may be an adaptive mechanism employed by quinoa to overcome NaCl stress (Figure 2). The hydrolysis of starch, protein, and lipid requires several essential endogenous hydrolases, and NaCl stress may influence the hydrolysis of these substances by altering the activity and gene expression of these enzymes, hence affecting the growth of quinoa.

Polyphenols are secondary metabolites produced during plant growth, particularly under adverse conditions. Salinity typically leads to an increase in the presence of phenolics and flavonoids in cells [34]. Grains contain polyphenols in both free and bound forms. Quinoa’s major phenolic acids include ferulic acid, *p*-coumaric acid, and vanillic acid, while its major flavonoids are quercetin, kaempferol, and rutin [35]. The present study found that exposure to NaCl stress at 50 mM and 100 mM resulted in a further increase in the levels of phenolics and flavonoids in MGQ (Figure 3). This finding is partially comparable to the results of a previous study [36], which showed an increase in the levels of phenolics and flavonoids in wheat under salt stress conditions. NaCl stress enhanced the phenolic content of MGQ, possibly due to the activation of the phenolic production pathway. In general, bound phenolics are stored in the plant cell wall, whereas soluble phenolics are mostly synthesized in the cellular endoplasmic reticulum via multiple routes, with glucose serving as the initial precursor [37]. The phenylpropane pathway is the principal mechanism for phenolic synthesis, with important enzymes such as PAL, C4H, 4CL, CHS, CHI, and CHR [38]. In this study, NaCl stress at 50 mM and 100 mM increased the activities of PAL, C4H, 4CL, CHS, CHI, and CHR as well as up-regulated the expression of genes related to the above enzymes (Figure 4 and Figure 5). This result is comparable to that of salt-stress-induced expression of genes and enzyme activity for phenylpropane metabolism in maize [39]. The increase in total phenolic content may attributed to enzymatic hydrolysis of bound polyphenols, which increases the content of extractable polyphenols [35]. As a result, NaCl stress may also further enhance the phenolic content of MGQ by stimulating the release of bound polyphenols.

Polyphenols are a class of antioxidants with a wide range of physiological activities and have demonstrated a strong free radical scavenging capability in antioxidant assays [40]. The increase in phenolic content is a significant contributing factor to the increase in antioxidant activity. DPPH and ABTS free radical scavenging capabilities are useful indicators for evaluating antioxidant activity in vitro [41]. Our work discovered that NaCl stress at 50 mM and 100 mM increased the scavenging capabilities of the DPPH and ABTS radicals to free phenols, bound phenols, and total phenols of MGQ (Figure 6). This finding is similar to that of 100 mM of NaCl stress, which increased DPPH and ABTS free radical scavenging capabilities in kiwifruit seedlings [42]. The results of increased DPPH and ABTS scavenging capabilities under NaCl stress were consistent with those of increasing phenolic content. NaCl stress at optimum concentrations may boost antioxidant activity by raising the content of other antioxidants such ascorbic acid and carotenoids. In addition, NaCl stress can generate a rapid ROS burst, resulting in oxidative damage to cells and inhibiting plant growth. Plants have mechanisms to cope with this injury, i.e., scavenging excess ROS by activating antioxidant enzymes including SOD, POD, CAT, APX, and GSH-Px to protect normal cellular metabolism [43]. In this study, NaCl stress at 50 mM and 100 mM activated SOD, POD, CAT, APX, and GSH-Px in quinoa (Figure 7). Enzyme activities of SOD, POD, CAT, and APX were also elevated under salt stress conditions in faba bean seedlings [44]. In contrast, a higher concentration of NaCl stress reduced the antioxidant capability and antioxidant enzyme activities of MGQ, potentially causing irreversible oxidative damage to quinoa cells and eventually inhibiting quinoa growth.

## 5. Conclusions

NaCl stress inhibited the growth of MGQ but contributed to its phenolic synthesis and antioxidant system. NaCl stress inhibited the hydrolysis of starch, protein, and fat, which may have led to the inhibition of MGQ growth. Lower concentrations (50 and 100 mM) of NaCl stress increased phenolic content. This was due to the fact that NaCl stress further increased the enzymatic activities of PAL, C4H, 4CL, CHS, CHI, and CHR, as well as gene expression. In addition, NaCl stress enhanced the DPPH and ABTS scavenging capacities and increased the activities of antioxidant enzymes such as SOD, POD, CAT, APX, and GSH-Px, enhancing the antioxidant system. Regarding this, 100 mM of NaCl stress had the best combined effect on MGQ. The synergistic application of magnetic field and NaCl treatments exerted salutary influences on the growth and secondary metabolic profiles of quinoa, with notable enhancements in the biosynthesis of phenolic compounds. Utilization of this treatment modality could elevate the nutritional content and antioxidant properties of crops, subsequently augmenting their commercial worth and delivering substantial health advantages to consumers. However, the response of quinoa to magnetic field and NaCl treatments may be affected by other factors. Therefore, more detailed studies on different quinoa varieties, treatment times, and environmental conditions are needed to maximize the effect of the combined treatment.

## Figures and Tables

**Table 1:** List of primer sequences used for quantitative real-time PCR analysis.

Gene	Access No.	F(5′-3′)	R(5′-3′)	Length (bp)
*PAL*	XM_021904443.1	TCGGTAGAGCTCGCTGAGT	AATACTCCAGCGTTCAAAAATCTTA	188
*C4H*	XM_021861074.1	ATTGATCACATTCTGGAAGCACAAG	TAGCTCGTCCCTTAGCTTCCT	188
*4CL1*	XM_021868660.1	AAGACCACAAATAATCTCACCCAA	TTCCCAGAATCAGAGTCAATCAA	114
*4CL3*	XM_021900806.1	TCCAAGGTGGACGACTTAATCT	CTCGGTCTTCATCGGTAAAACTA	140
*4CL5*	XM_021896032.1	TTGGCTATGTAGATGATGACGATG	TGCTGGTGGAACCTGGAAG	89
*4CL6*	XM_021909854.1	CGGTGCTGCCCCTTTAACTA	TCAGTCATGCCATAACCCTGAA	164
*CHS*	XM_021906739.1	AGTTTAAGCGCATGTGTGACAA	TCCCATGTAAGTACACATGTTAGGA	125
*CHR*	XM_021892157	ACGAGCAATCCACCTTACAACT	AGTGCCTAAGCCGATGACG	105
*CHI1*	XM_021900156.1	ACCTCATGGATCTCTTACGATAGG	TACAGCCTCCGACAAGTTCT	161
*CHI2*	XM_021882243.1	AATGGAAGGGTAAATCAGGAAAG	CCACAGGTGCTGCGGTAAG	75
*Actin-1*	XM_021904392.1	ATGTTCCCTGGTATCGCTGA	TGATCTTCATGCTGCTGGGG	70

## Data Availability

The original contributions presented in the study are included in the article/Supplementary Material, further inquiries can be directed to the corresponding authors.

## Supplementary Materials

The following supporting information can be downloaded at: https://www.mdpi.com/article/10.3390/foods13203278/s1, Figure S1: Schematic diagram of the device for processing quinoa.
