# Supplementary material for: NaCl Stress Stimulates Phenolics Biosynthesis and Antioxidant System Enhancement of Quinoa Germinated after Magnetic Field Pretreatment
Source: Foods. 2024 Oct 16;13(20):3278. doi: 10.3390/foods13203278 (PMC11507989; doi:10.3390/foods13203278)
Supplement: Supplementary file 1 [file foods-13-03278-s001.zip › foods-3235279-supplementary.pdf]

Supplementary data for

NaCl stress stimulates phenolics biosynthesis and antioxidant system enhancement of quinoa germinated after magnetic field pretreatment

Shufang Wang[1, 2], Xuejiao Zhang[2, #], Yiting Wang[2], , Jirong Wu[1],

Yin-Won Lee[1, 3], Jianhong Xu[1,*], Runqiang Yang[2,*]

[1] Jiangsu Key Laboratory for Food Quality and Safety-State Key Laboratory Cultivation Base, Ministry of Science and Technology/Key Laboratory for Control Technology and Standard for Agro-product Safety and Quality, Ministry of Agriculture and Rural Affairs/Key Laboratory for Agro-product Safety Risk Evaluation (Nanjing), Ministry of Agriculture and Rural Affairs/Collaborative Innovation Center for Modern Grain Circulation and Safety/Institute of Food Safety and Nutrition, Jiangsu Academy of Agricultural Sciences, Nanjing, Jiangsu, 210014, China

[2] College of Food Science and Technology, Whole Grain Food Engineering Research Center, Nanjing Agricultural University, Nanjing, Jiangsu, 210095, China

[3] Department of Agricultural Biotechnology, Seoul National University, Seoul 08826, Republic of Korea

E-mail: Shufang Wang: wangshufang202301@163.com

Xuejiao Zhang: zhangxuejiao8@126.com

Yiting Wang: 9201810401@stu.njau.edu.cn

Jirong Wu: yangzhouwjr@126.com

Yin-Won Lee: lee2443@snu.ac.kr

Jianhong Xu: xujianhongnj@126.com

Runqiang Yang: yangrq@njau.edu.cn

*** Correspondences:**

Jianhong Xu (xujianhongnj@126.com); Runqiang Yang (yangrq@njau.edu.cn)

Tel/Fax: + 86-025-84392001/ 84396293

[#] Equal contribution with the first author.

The device consists of four parts: a Helmholtz coil (Testron HM-260, China), a circulating water bath, a treatment chamber, and a power supply (JP 10022D, ANS Co., Ltd, China). First, quinoa seeds were placed in the sample treatment chamber and exposed to SMF. The desired SMF intensity was obtained by adjusting the current in the solenoid coil and measured using a portable digital gauge (TD-8620, Tunkia Co., Ltd, China). The temperature was controlled at 25 °C by circulating a water bath during the exposure process.

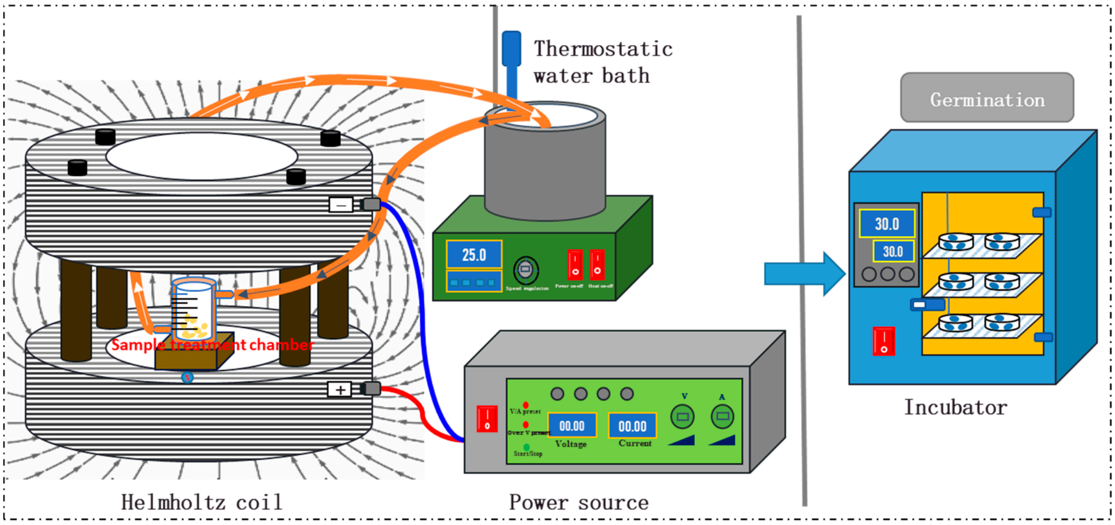

Figure S1. Schematic diagram of the device for processing quinoa.
